# Automatic detection of oral cancer in smartphone-based images using deep learning for early diagnosis

**DOI:** 10.1117/1.JBO.26.8.086007

**Published:** 2021-08-28

**Authors:** Huiping Lin, Hanshen Chen, Luxi Weng, Jiaqi Shao, Jun Lin

**Affiliations:** a Zhejiang University School of Medicine, First Affiliated Hospital, Department of Stomatology, Hangzhou, China; b Zhejiang Institute of Communications, College of Intelligent Transportation, Hangzhou, China

**Keywords:** deep learning, smartphone-based imaging, image collection, oral cancer diagnosis, oral potentially malignant disorders

## Abstract

**Significance:** Oral cancer is a quite common global health issue. Early diagnosis of cancerous and potentially malignant disorders in the oral cavity would significantly increase the survival rate of oral cancer. Previously reported smartphone-based images detection methods for oral cancer mainly focus on demonstrating the effectiveness of their methodology, yet it still lacks systematic study on how to improve the diagnosis accuracy on oral disease using hand-held smartphone photographic images.

**Aim:** We present an effective smartphone-based imaging diagnosis method, powered by a deep learning algorithm, to address the challenges of automatic detection of oral diseases.

**Approach:** We conducted a retrospective study. First, a simple yet effective centered rule image-capturing approach was proposed for collecting oral cavity images. Then, based on this method, a medium-sized oral dataset with five categories of diseases was created, and a resampling method was presented to alleviate the effect of image variability from hand-held smartphone cameras. Finally, a recent deep learning network (HRNet) was introduced to evaluate the performance of our method for oral cancer detection.

**Results:** The performance of the proposed method achieved a sensitivity of 83.0%, specificity of 96.6%, precision of 84.3%, and F1 of 83.6% on 455 test images. The proposed “center positioning” method was about 8% higher than that of a simulated “random positioning” method in terms of F1 score, the resampling method had additional 6% of performance improvement, and the introduced HRNet achieved slightly better performance than VGG16, ResNet50, and DenseNet169, with respect to the metrics of sensitivity, specificity, precision, and F1.

**Conclusions:** Capturing oral images centered on the lesion, resampling the cases in training set, and using the HRNet can effectively improve the performance of deep learning algorithm on oral cancer detection. The smartphone-based imaging with deep learning method has good potential for primary oral cancer diagnosis.

## 1 Introduction

Oral cancer is one of the most common cancers worldwide, with high mortality rates. According to the International Agency for Research on Cancer, there were an estimated 377,000 new cases of lip and oral cavity cancers in 2020, with nearly 177,000 deaths worldwide.^1^ Despite advances in oncology therapy, mortality rates for oral cancer remain high over the past few decades. A majority of oral cancer patients do not have access to timely, quality diagnosis, and treatment, especially in rural areas, resulting in poor survival rates. The overall 5-year survival rate of diagnosed oral cancer patients is around 50% and has varied by race and area,^2^ whereas survival rates as high as 65% have been reported in developed countries; in some rural areas, they can be as low as 15% depending on the affected part of the oral cavity. The 2020 Cancer Statistics Report from India estimated that 66.6% of patients suffering from head and neck cancer were diagnosed at the locally advanced stage.^3^ In short, patient survival rates and prognosis are severely compromised when oral cancer patients are diagnosed at more advanced stages^4^^,^^5^ so that enhancing early diagnosis could mean a significant rise in positive survival outcomes.^6^^,^^7^

As oral squamous cell carcinoma (OSCC) accounts for ∼90% of all oral cancer,^8^^,^^9^ these two terms tend to be used interchangeably. OSCC, which originates as an epithelial dysplasia (from a histopathologic perspective), generally develops from precursor lesions termed as oral potentially malignant disorders (OPMDs).^10^^,^^11^ Nevertheless, it is not inevitable that all OPMDs, even the most commonly encountered such as oral lichen planus, leukoplakia, and erythroplakia,^11^ result in the subsequent development of malignancies.^10^^,^^12^ Diagnosing OPMDs as definable diseases is also challenging due to the numerous varieties, various forms, and overlapping features. However, studies^10^^,^^13^^,^^14^ have found that when an OPMD changes to a nonhomogeneous presentation, it is more likely to be considered as an adverse progression, in other words, nonhomogeneous lesions have a greater risk of malignant transformation as against homogeneous lesions. Hence, compared with defined diagnosis, distinguishing the potential malignant characteristics of OPMD is a greater concern. In summary, an ideal clinical prediction method should be employed to diagnose OSCC early and in particular to assess malignancy at the OPMD stage.

Currently, conventional oral examination (COE) consisting of visual and tactile assessment (followed by tissue biopsy if there are any suspicious findings) is the most routine procedure in the management of oral cancer and precursor disease. However, one limitation for COE is that several features of oral cancer may appear benign and even mimic aphthous ulcers, and they are too clinically heterogeneous and subtle for general dentists to distinguish. Second, because of its invasive nature and sampling bias that can lead to underdiagnosis or misdiagnosis, biopsy is often not ideal as a screening tool.^15^ Furthermore, although specialists can recognize most of the characteristics that differentiate benign and cancerous lesions, the number of specialists and health resources are limited and concentrated across regions, causing a large part of the oral cancer burden to fall on low-resource communities. Therefore, the idea of establishing a cost-effective screening strategy as an adjunctive aid to the current procedures is gaining widespread popularity.^16^

Recently, deep learning techniques have exhibited a comparative advantage over feature-based methods in medical image analysis. A variety of studies^17^^,^^18^ showed that deep learning algorithms are able to surpass the performance of human experts in many disease recognition scenarios. In oral cancer diagnosis, deep learning methods also showed promising results for automatic analysis of pathology, confocal laser endomicroscopy (CLE) images, and fluorescence images. For example, Kumar et al.^19^ proposed a two-stage method that used a segmentation network and a random forest tree classifier to identify different stages of oral cancer in histological images. Aubreville et al.^20^ tested the deep convolutional neural network for OSCC diagnosis on CLE images, and the results showed that it outperformed the feature-based classification methods. Song et al.^21^^,^^22^ developed mobile connected devices to acquire fluorescence oral images and used them to identify oral disease. However, these methods all require expensive devices or a specifically designed screening platform, which are not accessible to everyone. In other words, patients are still required to go a professional clinic to receive disease diagnosis.

With the rapid development of both imaging and sensing technologies in camera systems, the ubiquity of smartphones is equipped with higher quality, low-noise, and faster camera modules. Smartphone-based white light inspection methods^23^^–^^25^ are good solutions for acquiring oral images. Camalan et al.^23^ used a CNN-based network for classifying white light images as normal or suspicious; however, the patient sample is very limited: with only 54 cases. To build a reliable system, Welikala et al.^24^ collected more clinically labeled data for network training and evaluation, achieving a sensitivity of 52.13% and a specificity of 49.11% on multiclass classification task. However, we found that the clinical imaging capturing from hand-held smartphone cameras may exhibit a large variability, leading to poor diagnostic performance of the detection algorithm. For example, from a computer vision point of view, the shape and size of imaging lesions depend on the fields-of-view and focal distance, respectively. Unfortunately, a limited amount of research focuses on this problem. To address these challenges, we aim to explore reliable and robust smartphone-based white light image approaches, including an image-capturing method, resampling method, and high performance of CNN model, for oral disease recognition. Our main contributions are summarized as follows.

1. We propose a simple yet effective image-capturing method for consistent lesion position and focal distance over different images. The method allows direct focus on discriminative parts for disease recognition, without utilizing any region proposal methods,^24^^,^^25^ or relying on bounding box annotation^24^ by oral specialists.
2. We present a resampling method to alleviate the effect of image variability that introduced by the hand-held smartphone camera; at the same time, it can remedy the class imbalance problem.
3. We use one of the latest proposed convolutional neural networks (HRNet) for oral disease classification and achieve better results than common classification models. Analysis of the model’s performance on our collected images shows that common imaging pattern on a smartphone is a valuable approach for the early diagnosis.

## 2 Materials and Methods

### 2.1 Image-Capturing Method

The focal length of the main camera in a smartphone is commonly short, for example, the iPhone 12 has a wide-angle (only 26 mm) camera. The focal length number tells us how much of the scene is captured in the picture, and the lower the number is, the wider the view is. Since most lesions are relatively small, we cannot capture the photograph where the lesion occupies most areas of the image. This means that the captured image may have many irrelevant backgrounds. In addition, even with the same lesion, the size of the imaging lesion may vary with different distances between the camera and lesion or using different cameras with different focal lengths. Thus these would introduce the large variability to the system’s performance going forward, and we need to find a method to reduce this considerable variability.

Although identifying oral disease is very difficult for the nonmedical expert, locating the position of oral lesions is relatively easy because the visual appearance of normal and diseased tissue is significantly different. We use the camera grid to assist in locating the lesion in the center of image and to keep each area of lesion in the images neither too small nor too big. Thus it is possible to use the fixed region of interest (ROI) method to crop the discriminative parts and filter the irrelevant backgrounds, without utilizing any region proposal methods or relying on any manually cropped methods. This particular positioning of the main object in an image is helpful to improve the performance of CNN for image recognition.

**Fig. 1:**
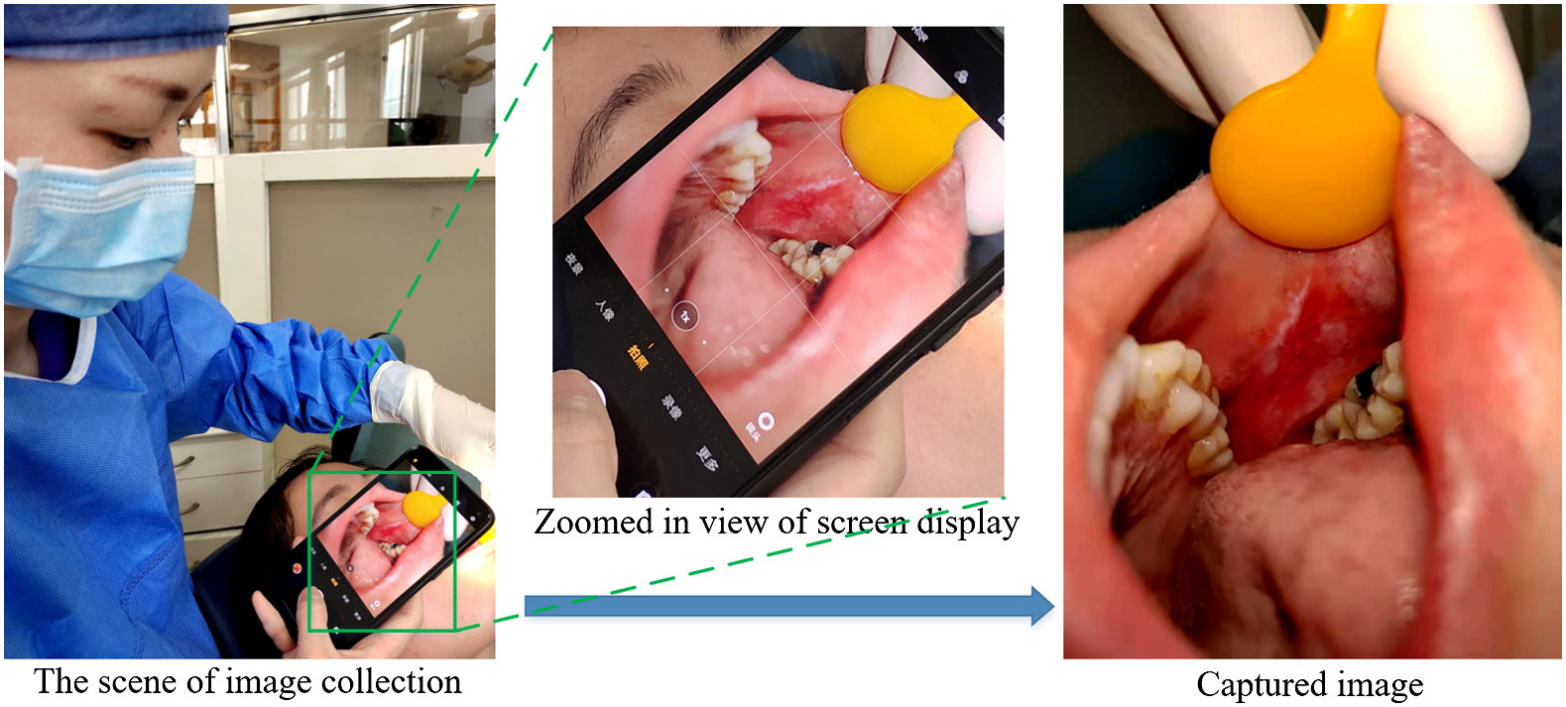
Illustration of data acquisition. The aspect ratio is set 4:3; the image shows a lesion at the center of the region.

We use the native phone’s camera app to capture the oral cavity image. As shown in Fig. 1, the camera grid helps us to see if the lesion is properly placed at the center for optimal balance in the shot. The operation can be easily done by a person using a hand-held smartphone camera. The basic steps are as follows.

1. Turn on the camera grid within the settings. This will display a faint grid over the capture frame, split into nine rectangles of equal size.
2. Take the image from the patient, who has given informed and approved consent. Hold the phone camera near the lesion, keeping the lesion in the center of the square, and then control the distance between the camera and lesion to keep the covered lesion area (showing in the camera screen) that is slightly smaller than the center camera grid region. Then press the shutter to capture the lesion image.
3. Upload the captured image to a computer with diagnostic software, via a direct line connection or WiFi.

### 2.2 The Oral Dataset

**Fig. 2:**
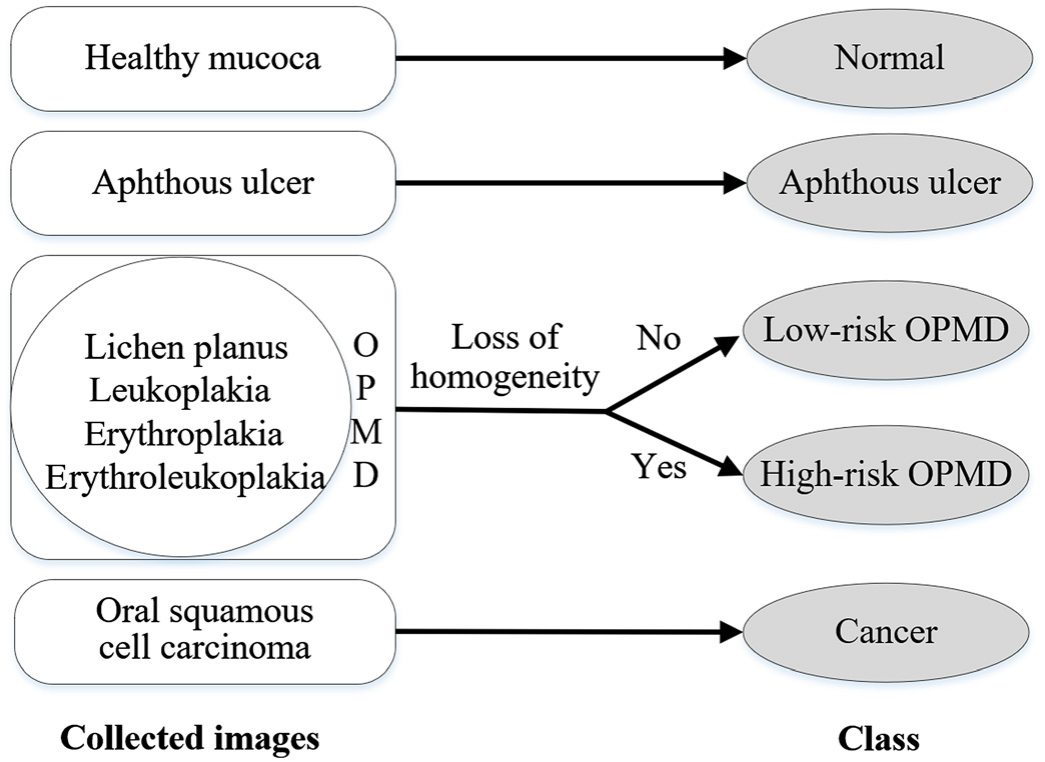
Overview of the five categories based on initial clinical impression.

This study is approved by the Medical Research Ethics Committee of the First Affiliated Hospital, College of Medicine, Zhejiang University, China. Image collection is carried out in the daily outpatient clinics and on inpatients of the hospital. We use four different smartphones, including iPhone 11, iPhone 12, IQOO U1, and 360 N7Pro, to retrospectively collect oral images from subjects over 18 years of age, including healthy people and those who are clinically diagnosed with aphthous ulcers, OPMD, or oral cancer. Because loss of homogeneity is a key visual feature indicating potential carcinogenesis, a binary system is employed for OPMD, which divides lesions into “low risk” (homogeneous) and “high risk” (nonhomogeneous) on the basis of clinical manifestations. Therefore, all the oral images fall into one of five categories based on initial clinical impression, namely, normal, aphthous ulcer, low-risk OPMD, high-risk OPMD, or oral cancer, as shown in Fig. 2.

**Fig. 3:**
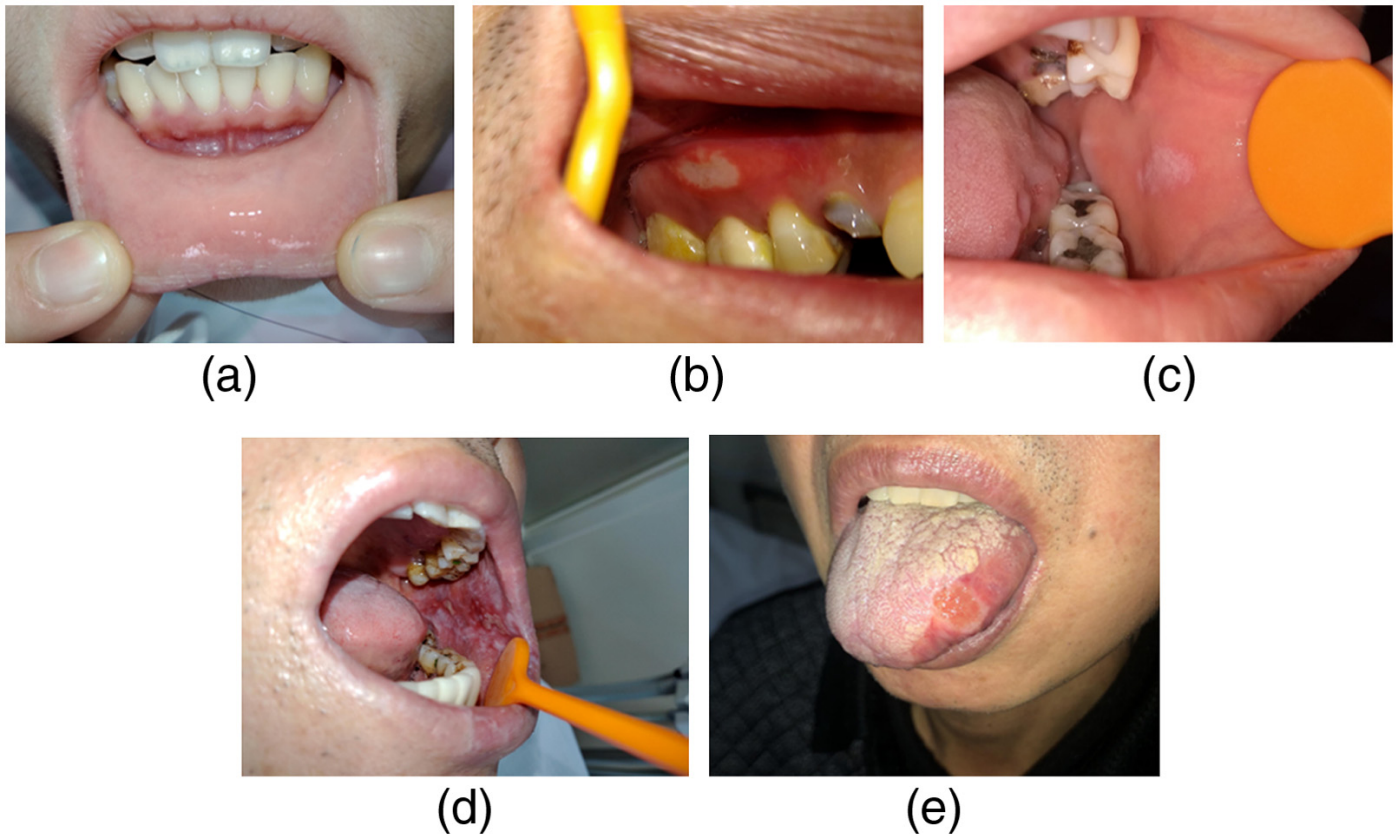
Clinical features of sample images in the five categories. (a) Normal mucosa image from lower labial mucosa and upper lip of a healthy subject. (b) Aphthous ulcer on the attached gingiva. (c) A homogeneous white patch on the left buccal mucosa. (d) An extensive white lesion with a red component affecting the whole left buccal mucosa. (e) A malignant lesion on the lateral border of the tongue.

Different clinical features of examples in the five categories, shown in Fig. 3, are described below. The healthy mucosa presents as homogeneous, pink, and shiny, with neither white nor red patches. Aphthous ulcer lesions clinically describe shallow, round, or ovoid ulcers covered with a yellow pseudomembrane and surrounded by an erythematous halo. They may appear on any site of the oral cavity. OPMD refers to a wide range of definable diseases but has confusing clinical appearances. Low-risk (homogeneous) OPMD often presents as white patches, which are uniformly flat and thin and have a smooth or fissured surface without any atrophic or erosive lesions. For high-risk OPMD, the most important difference from homogeneous OPMD is the existing red components mixed into white patches, making the lesion either atrophy or erode with irregular surface texture (nonhomogeneous). Oral cancer is often shown as a nodular lesion protruding from the mucosal surface or a deep ulcer with a rough surface, both with unclear boundaries.

One image is taken for each lesion instance, in another words, if one patient contains multiple oral disease instances, we will take multiple images. Otherwise, we only collect one oral cavity image from each patient. The statistics of the number of images, the number of patients, images per patient, and images per smartphone type can be seen in Table 1. The normal cases can be taken several images from the different anatomic sites of oral cavity, including tongue (dorsal or ventral), palate, labial mucosa (upper or lower), buccal mucosa (left or right), and the floor of the mouth, in a healthy person. In addition, the patients with low-risk or high-risk OPMD are more likely to have multifocal lesions compared to those in the aphthous ulcer group or cancer group.

**Table 1:** Statistics of patient and smartphone characteristics in our oral disease dataset.

Statistic	Normal	Aphthous ulcer	Low-risk OPMD	High-risk OPMD	Oral cancer
Number of images	760	251	231	141	65
Number of patients	232	218	165	107	65
Image per patient (mean)	3.28	1.15	1.40	1.32	1.00
Smartphone type:					
iPhone 11	87	21	0	5	27
iPhone 12	114	27	16	4	12
IQOO U1	212	58	63	45	15
360 N7Pro	347	145	152	87	11

**Fig. 4:**
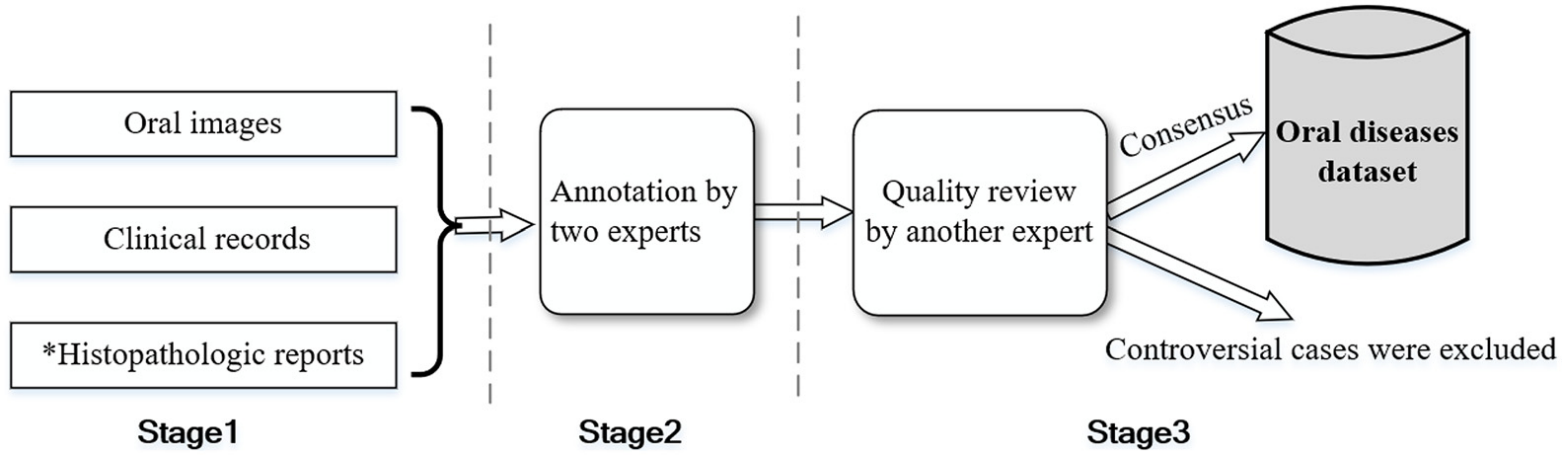
Schematic flow of data annotations from multiple experts. *Not all cases have a histopathologic report.

For ensuring a high quality of annotation, we invite three oral medicine specialists, who are committed to the preventive treatment of oral cancerous and potentially malignant disorders, to manually annotate images. Figure 4 illustrates the schematic flow of data annotations. First, we collect the lesion images from the patients and access their clinical records (including health records and medical history) as well as histopathologic reports if a subsequent biopsy is performed. All patients initially diagnosed with oral cancer have undergone histopathologic examination. The cases with histopathologic reports can be seen as the gold standard. Other mild symptomatic lesions, such as aphthous ulcer and low-risk OPMD, may not have a histopathologic report. Next, each image receives annotations from two separate individual senior experts (an average of 30 years of clinical practice), with reference to the appearance of symptoms and clinical records or histopathologic reports. Finally, another expert who specializes in mucosal diseases with 9 years of clinical experience, reviews each case label to confirm the initial assessment, and the cases labeled as containing multiple oral disease conditions or as controversial by the experts are excluded from the dataset.

The final oral data contains 688 oral lesion images and 760 normal mucosa images. Overall, the images are derived from a wide range of people between 18 and 82 years of age and appeared in a variety of anatomic sites. Table 2 summarizes the number of images acquired for different anatomic sites in the dataset. The 688 lesion images included images of aphthous ulcer (251), low-risk OPMD (231), high-risk OPMD (141), and cancer (65). The dataset is divided randomly into two subsets based upon the different patients and restricted the proportion of the number of each class. Considering that our study mainly focuses on early diagnosis of oral cancer, and the high-risk OPMDs and cancers are harmful types of disease than that of others in the dataset, we set higher split ratios of these two classes in the test set to make the experimental results more convincing. The test set (455 cases) consists of 228 normals (30% of normal), 76 aphthous ulcers (30%), 69 low-risk OPMDs (30%), 52 high-risk OPMDs (37%), and 30 cancers (46%).

**Table 2:** Overview of image numbers for different anatomic sites in our oral disease dataset.

Site	Normal	Aphthous ulcer	Low-risk OPMD	High-risk OPMD	Oral cancer
Lip and labial mucosa	75	114	4	7	7
Buccal mucosa	314	35	147	93	13
Gingive and alveolus	36	17	22	9	11
Tongue	136	56	41	28	23
Palate	119	13	16	3	11
Floor of the mouth	80	16	1	1	0
Total	760	251	231	141	65

### 2.3 Data Processing

**Fig. 5:**
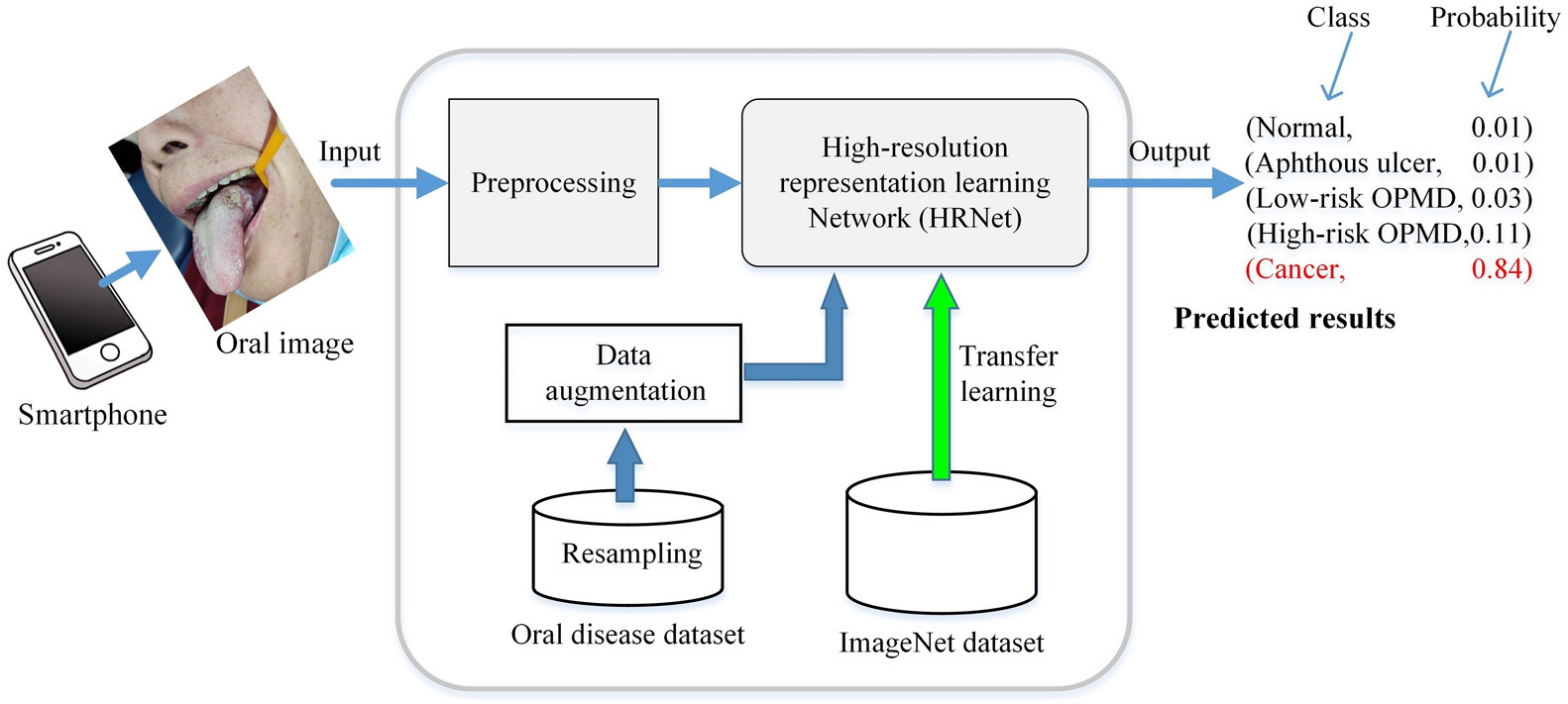
Overview of the proposed method for classifying oral disease images.

For each case, our CNN-based system takes one oral image as input and outputs a set of the probability of each disease. The final prediction is made based on the class with the highest confidence score. To make the research easier for end user, we developed custom diagnostic software written in Python and implemented using open-source PyQT and PyTorch libraries. Our CNN-based system involves three steps as shown in Fig. 5. The first step is to capture the patient’s lesion with a smartphone, creating a digital image following the previously described method (see Sec. 2.1). This can also easily be done by the patient with the help of other people, using a hand-held smartphone camera. The images could contain irrelevant objects, so it is necessary to extract the useful information in the next step.

The following step is preprocessing. The center-area cropping is used to obtain ROI image. The identification of an ROI generally requires looking at areas of the image near the lesion for additional information, and this holds true for both human experts and deep learning algorithms. Thus we set the ROI to be larger than the center grid region. Also data normalization that modified the pixel intensity and contrast of the image is used in preprocessing. Specifically, for each pixel in the input image, we first convert it from the range [0, 255] to a range [0, 1.0]. Then for each channel of the input, one subtracts the corresponding channel mean and then divides by corresponding channel std. To reduce the computational cost, we resize the ROI images to 512×512  pixels for the following process.

The last step is image classification based on CNN technology. The recently introduced high-resolution representation learning network (HRNet), which is pretrained on the ImageNet^26^ and then fine-tuned all the weights on our oral dataset (transfer learning), is used to analyze the images and output the relative likelihood of the five categories.

In the training stage, we use the proposed resampling method to expand the training set. The details can be found in the next section.

### 2.4 Resampling

To alleviate the effect of variability in images that were collected from our image-capturing method, we propose the resampling method. The resampling process is only performed on the training set.

**Fig. 6:**
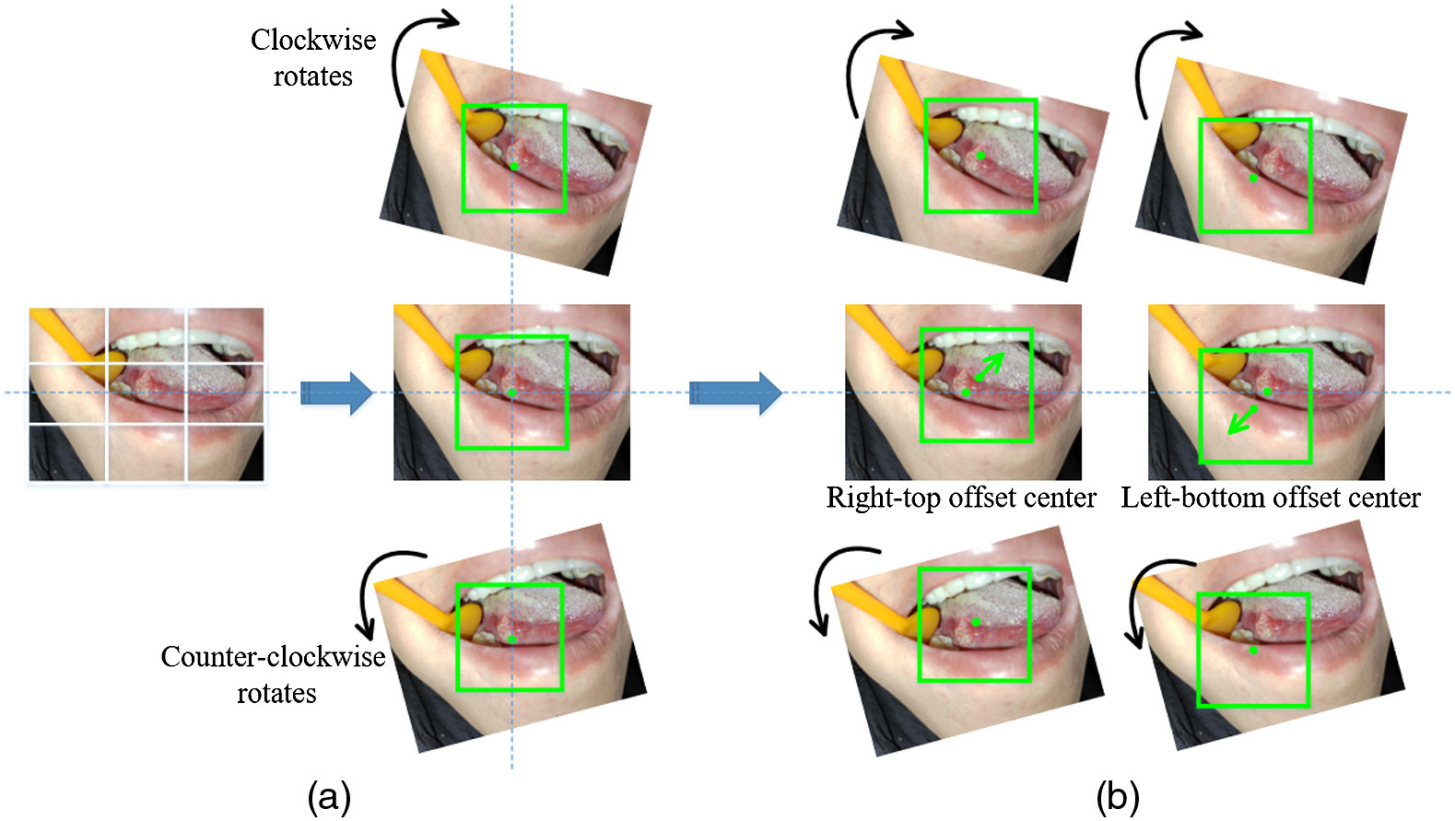
The captured image is expanded by an optional offset center of rotation. The green squares mark the cropped areas that will be used to create patches for training. Rotate the image around (a) its center and (b) its offset center.

Although the above image-capturing method is roughly centered on each lesion, the oral images may be captured from slightly different angles. We use image rotation to compensate for this. Specifically, two-direction rotations with one of a step size of θ deg and another a step of −θ  deg are performed on each oral image, as shown in Fig. 6(a). Then we crop images to a fixed size that included only the lesion and sufficient discriminative information. We set θ=15 in the experiments. Accordingly, three patches of size H×W centered at the image centers are extracted as training samples, and the number of samples in each class is 1596, 525, 486, 267, and 105.

Another problem is that the captured lesion center may be inaccurate in practice, we move each lesion centroid $N_{t}$ times to obtain $N_{t}$ points as the approximate nucleus, as shown in Fig. 6(b). Then we use the above method to extract the local neighboring regions again, and thus further increase our number of image samples. Considering the imbalance of sample sizes in datasets (for example, we collected about 5 times the number of ulcer samples compared to cancer samples), training deep networks with this data can induce a positive bias in the large rate class. To compensate class imbalance, there are many solutions, such as using cost-modifying methods^27^^,^^28^ to balance the learning and introducing GANs^29^ to generate data scarcity samples. Here we set different values of $N_{t}$ to handle unbalanced data and center location bias at the same time. It should be mentioned that we only perform rotation on the normal samples and skipped the offset resampling operation. For the ulcer, low-risk OPMD, high-risk OPMD, and cancer samples, we set $N_{t}$ as 3, 3, 5, and 15, respectively. The final training set consists of 1596 normals, 1575 aphthous ulcers, 1458 low-risk OPMDs, 1335 high-risk OPMDs, and 1575 cancers.

The proposed resampling method has two main differences from the common data augmentation that increases the number of samples during the training process. First, the standard rotating augment approach, feasible at training, will result in zero-padding or image size change, whereas our method does not suffer this problem. Second, in our resampling method, the expanded number of each class of samples is controllable by setting a propertied rate of $N_{t}$ and offset angle θ.

### 2.5 Network Architecture and Classification

In this study, we treat the problem of oral disease diagnosis as a multiclass classification. Let $x_{i}\in R^{H\times W\times 3}$ be an input image containing a lesion to be classified. The predicted class $c_{i}$ for input image $x_{i}$ with corresponding output $F(x_{i})$ from HRNet-W18 is given as

$$c_{i}=\mathrm{argmax}\;p_{j}(F(x_{i})),$$ (1)

where $p(F(x_{i}))$ is a softmax function defined by

$$p(F(x_{i}))=\frac{\exp(F(x_{i}))}{\sum_{k}\;\exp(F(x_{i}))}.$$ (2)

**Fig. 7:**
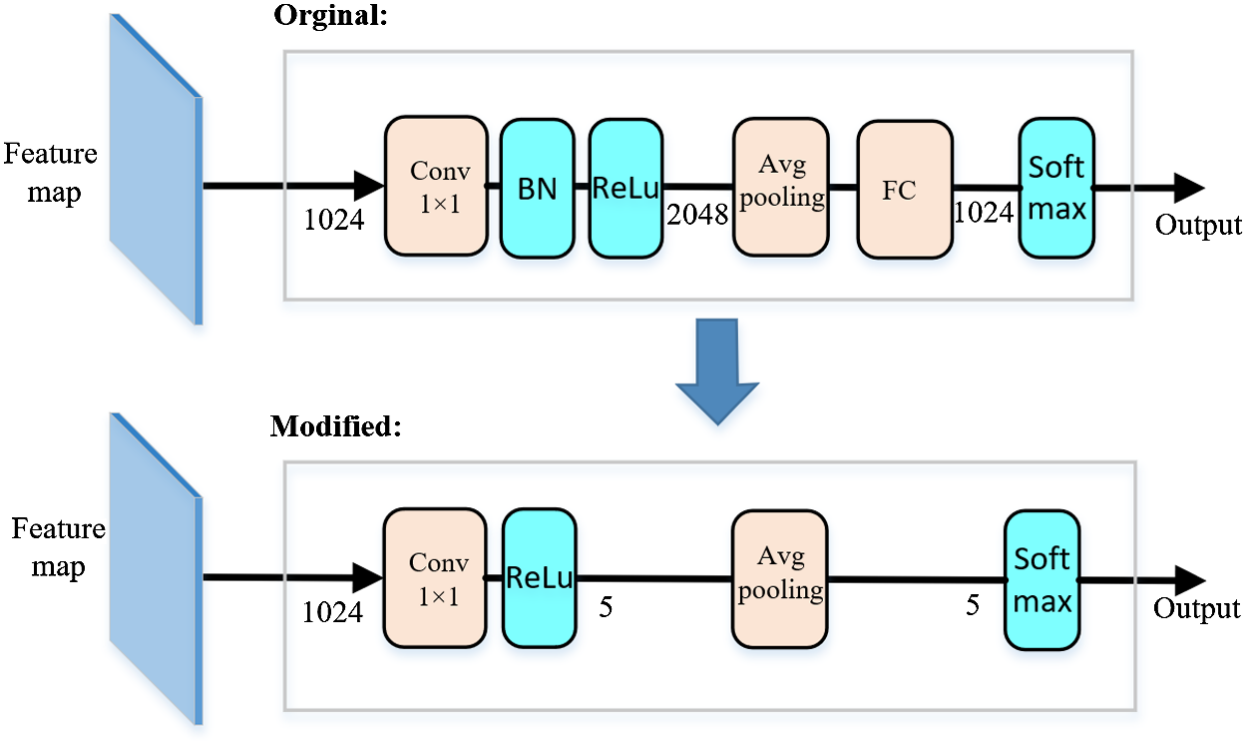
Illustration of modified representation head in HRNet-W18. The number in each layer is the number of channels. BN is the abbreviation of “batch normalization,”^30^ FN is the abbreviation for “fully connected.”

Recently, high-resolution representation learning has shown great potential in various vision applications, and related high-resolution networks (HRNet V1, HRNet V2, etc.)^31^^,^^32^ have been proposed. HRNet utilizes high-resolution representation learning to maintain semantic richness and spatial precision. The results reported by authors are slightly better than existing networks such as ResNet50,^33^ VGG16,^34^ or DenseNet169^35^ for ImageNet classification. Therefore, we use the HRNet-W18 as the CNN for disease diagnosis; W18 represents the widths of the high-resolution subnetworks in the last three stages, respectively. The HRNet-W18 takes images of the oral cavity as input and outputs probability of disease positivity. To simplify the structure of the network and reduce the computational cost, we follow the network in network strategy^36^ to modify the representation head of the HRNet-W18. Specifically, we decrease output of the channel of the 1×1 convolution layer from 2048 to 5. Then we use a global avg-pooling layer that directly outputs log probability (instead of the design that uses an average-pooling layer followed by a fully connected layer as the last layer) as shown in Fig. 7.

## 3 Results

### 3.1 Network Training

The computing hardware was a desktop computer with i5-8400 CPU, 24G RAM, and a single Nvidia GeForce GTX3090 GPU. The HRNet-W18 was written in Python, built on the PyTorch,^37^ and run on the Ubuntu18.04 operating system. HRNet was trained under the supervised learning method that minimizes the error between predicted probability and the true class labels. A common cross-entropy loss function was used for the supervised learning. To speed up the training and improve training performance, we used the preinitialization from ImageNet dataset. We did not freeze any parts of the layers and retrained the whole network. The initial learning rate was set to 0.001 and was reduced by 30% at epoch 15, 30, and 45. We utilized SGD^38^ with a weight decay of 0.0001 and a Nesterov momentum of 0.9. HRNet-W18 training was applied with a fivefold cross-validation strategy on the training set and stopped after a fixed number of epochs (100) with a mini-batch size of 24. We selected the single best-performing network on the hold-out validation set as the final model for testing.

Although the collected cases were used to generate more training data by the resampling method (described in Sec. 2.4), the training samples were still limited, making the deep learning algorithm more prone to overfitting and not well generalized to new examples. Data augmentation has been demonstrated to be an effective method for improving the performance of CNN with a limited training sample.^39^^,^^40^ We also applied common data augmentation, including left–right flipping, random resize cropping with ratio 0.7 to 1.0, arbitrary brightness, and contrast with ratio 0.9 to 1.1, and, for the model training. In testing, we did not use any data augment methods as they do not have a significant effect on prediction. Only a single forward pass on each image was performed to obtain the final score, which makes the approach potentially more efficient than the ensemble approaches.

### 3.2 Evaluation Metrics

For the multilabel classification problem, we used the macroaverages of sensitivity, specificity, precision, and F1 metrics to evaluate the performance of the models. We first measured the prediction errors for each separate class and then reported the final scores by averaging these results weighted equally. Let C be the number of classes, those metrics are defined as

(i) ${\mathrm{SE}}_{\mathrm{mro}}=\frac{1}{C}\overset{C}{\underset{i=1}{\sum }}{\text{sensitivity}}_{i}$, ${\text{sensitivity}}_{i}=\frac{{\mathrm{TP}}_{i}}{{\mathrm{TP}}_{i}+{\mathrm{FN}}_{i}}$,
(ii) ${\mathrm{SP}}_{\text{macro}}=\frac{1}{C}\overset{C}{\underset{i=1}{\sum }}{\text{specificity}}_{i}$, ${\text{Specificity}}_{i}=\frac{{\mathrm{TN}}_{i}}{{\mathrm{TN}}_{i}+{\mathrm{FP}}_{i}}$,
(iii) ${\mathrm{Pr}}_{\text{macro}}=\frac{1}{C}\sum_{i=1}^{C}{\text{precision}}_{i}$, ${\text{precision}}_{i}=\frac{{\mathrm{TP}}_{i}}{{\mathrm{TP}}_{i}+{\mathrm{FP}}_{i}}$,
(iv) $F_{1}=\frac{2\times {\mathrm{SE}}_{\text{macro}}\times {\mathrm{Pr}}_{\text{macro}}}{{\mathrm{SE}}_{\text{macro}}+{\mathrm{Pr}}_{\text{macro}}}$,

where ${\text{sensitivity}}_{i}$ is the correct detection ratio of true positives in class i, ${\text{specificity}}_{i}$ is the correct detection ratio of true negatives in class i, and ${\text{precision}}_{i}$ is the proportion between the true positives and all prediction positives in class i.

### 3.3 The Effectiveness of the Image-Capturing Method and Resampling Method

To demonstrate the effectiveness of our image-capturing method, we compared the performance of the same two networks, in which one trained on images with centered lesions (our collected oral dataset), and the other trained on the images without prerequired lesion centering (random positioning). The latter images are from another dataset, which we created from the original dataset by a simulated image-capturing method that did not require lesion centering. Specifically, we cropped each original image at a random location to create a single smaller image, resulting in a wider range of lesion positions in the images; then we resized the cropped images to the size of 512×512 for network training and testing. We trained and tested the HRNet-W18 on the two datasets and the results are reported in the first two rows of Table 3. The model trained on the centered rule dataset achieved about 8% higher F1 score than that trained on the random positioning dataset. The reason behind this is that our image-capturing method can localize the ROI (the lesion region), and using the center cropping method, the CNN can focus on the discriminative regions rather than the whole cavity image that may have irrelevant objects, leading to better performance. This demonstrates that the proposed image-capturing method is effective for this oral disease detection task.

**Table 3:** Comparison of classification performance with different methods.

Method	${\mathrm{SE}}_{\text{macro}}$	${\mathrm{SP}}_{\text{macro}}$	${\mathrm{Pr}}_{\text{macro}}$	F1
Random positioning	0.717	0.938	0.753	0.730
Center positioning	0.786	0.954	0.790	0.787
Center positioning + over-sampling	0.776	0.954	0.791	0.778
Center positioning + resampling	**0.830**	**0.966**	**0.843**	**0.836**

Note: The values shown in bold represent the highest performance.

To qualitatively evaluate the effectiveness of the resampling method, we first used the random over-sampling method^41^ to produce more small class samples to keep the training class balance. On the other hand, we used the proposed resampling method to expand the training set. We retrained the HRNet-W18 on these two training sets and the test results were shown in the third to fourth rows of Table 3. We observed that the over-sampling method decreases the model’s performance since it is susceptible to over-fitting of the minority classes^42^^,^^43^ while our resampling method has an improvement of about 6% in F1 score. The results demonstrate the effectiveness of resampling method to boost the performance of the network on oral disease diagnosis.

### 3.4 Comparison with Other Networks

We evaluated and compared a number of neural network architectures, including VGG16, ResNet50, DenseNet169, and HRNet-W18 on our collected dataset. The results are reported in Table 4. In terms of the number of model parameters, HRNet-W18 has fewer parameters than VGG16 or ResNet50 but more than DenseNet169. Although the annotated training data was limited, all network architectures showed good performance on this dataset. We observed that HRNet-W18 achieved a sensitivity of 83.0%, specificity of 96.6%, precision of 84.3%, and F1 score of 83.6%. It outperformed other models in all metrics except specificity, which were the nearly same as for the DenseNet169. This comparison reflects that the effectiveness of high-resolution representations learned by the modified HRNet-W18 network in oral disease classification.

**Table 4:** Comparison of classification performance with different methods on the oral disease dataset.

Method	Params	${\mathrm{SE}}_{\text{macro}}$	${\mathrm{SP}}_{\text{macro}}$	${\mathrm{Pr}}_{\text{macro}}$	F1
VGG16	568M	0.728	0.950	0.767	0.745
ResNet50	24M	0.770	0.954	0.788	0.771
DenseNet169	**12M**	0.810	0.965	0.835	0.817
HRNet-W18	17M	**0.830**	**0.966**	**0.843**	**0.836**

Note: The values shown in bold represent the highest performance.

**Fig. 8:**
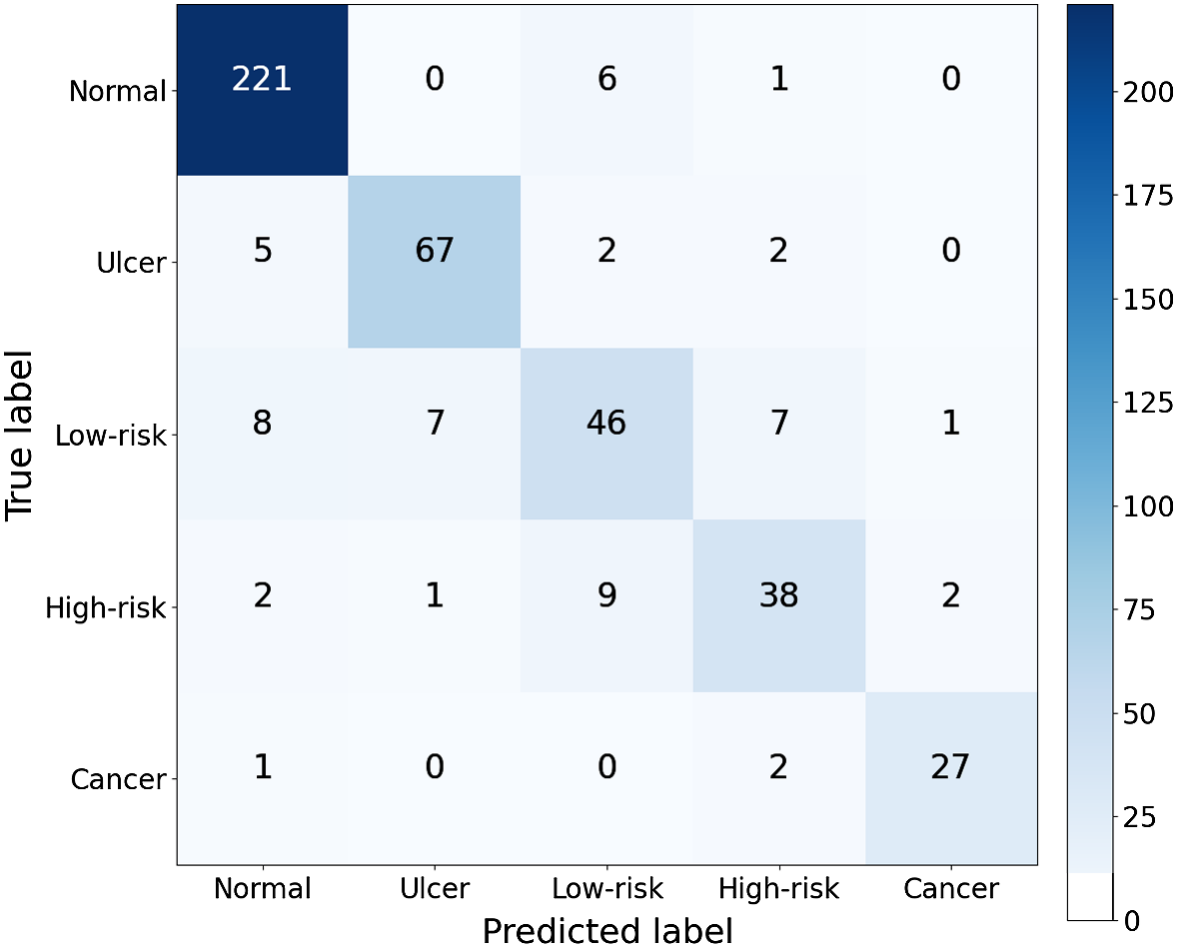
Confusion matrix for multiclass performance for the proposed method. The numbers of correct classifications are shown in the diagonal. The numbers of wrong classifications due to overdiagnosis and underdiagnosis are found in the top-right triangle and bottom-left triangle, respectively.

The confusion matrix in Fig. 8 was generated by applying the HRNet-W18 model to the test set. It reported the number of correctly classified cases and misclassified cases over the five classes. The performance of each class is listed in Table 5. The results showed F1 of 95.0% on the normal class, compared with the lower F1 scores for other classes, highlighting the fact that classes with scarce samples are generally more problematic to classify. In addition, we noticed that the proposed method has relatively higher error rates for low-risk and high-risk lesions than on the other classes. The results are understandable since the appearances of these two diseases are very similar, and it is also difficult to distinguish by less experienced dentists.^46^

**Table 5:** Diagnostic multiclass performance of the proposed method. The corresponding AUCs were computed in each case using one versus other strategy.^44^ The confidence intervals (CIs) of AUC at 95% were computed by the DeLong method.^45^

Class	AUC (95% CI)	F1
Normal	0.949 (0.929 to 0.970)	0.951
Ulcer	0.930 (0.907 to 0.953)	0.887
Low-risk	0.811 (0.766 to 0.857)	0.697
High-risk	0.851 (0.808 to 0.893)	0.745
Cancer	0.946 (0.923 to 0.970)	0.900

### 3.5 Visualization

**Fig. 9:**
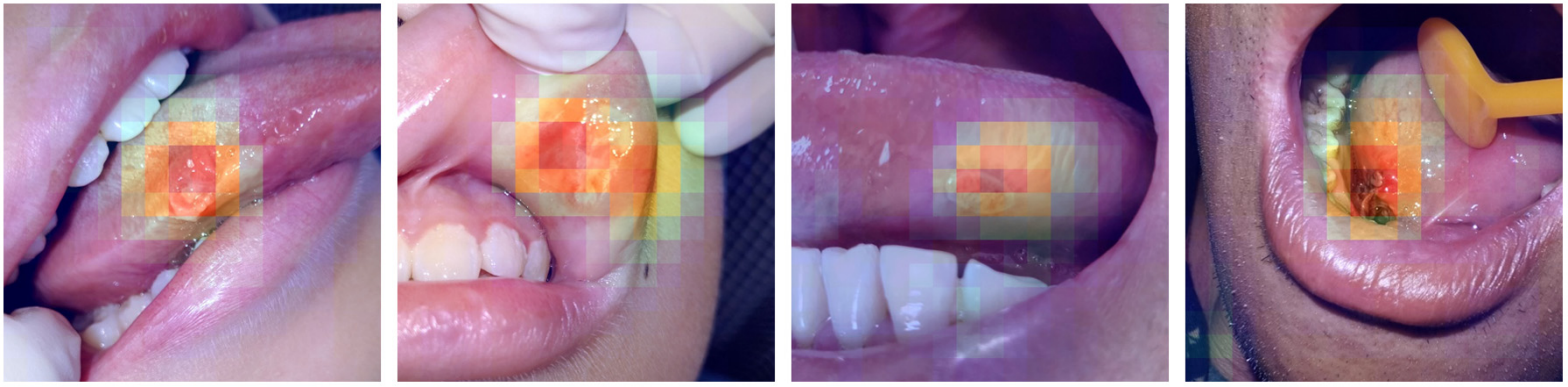
Examples of oral images with visualization. The heatmaps highlight the class-discriminative regions used for oral classification.

To better understand the CNN diagnosis, we introduced the class activation mapping^47^ technique to our HRNet-W18 for prediction visualization. We first removed the last layer (average-pooling layer) of HRNet-W18 and then upsampled the output’s class activation maps (by a 16-fold linear interpolation) to the original image size. The highlighted regions of the images showed that the regions were “important” for predictions from the model. As shown in Fig. 9, most of the lesion regions were correctly localized, illustrating that the final diagnoses are based on identifying the extent of objects.

## 4 Discussion

**Fig. 10:**
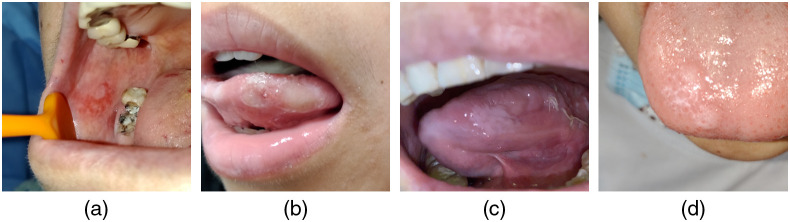
Representative examples of failure cases: (a) high-risk OPMD misclassified as an ulcer lesion; (b) ulcer lesion misclassified as high-risk OPMD; (c) high-risk OPMD misclassified as being cancerous; and (d) low-risk OPMD misclassified as normal.

We show some failure cases in Fig. 10. The high-risk OPMD case [Fig. 10(a)] is wrongly identified as an ulcer. Although the percentage of this misclassification by the model is low (only 1.9%), such missed cases should be avoided as incorrect recognition could lead to misdiagnosis, missing the optimal treatment window, or even to fatality. Figure 10(b) provides an example of an ulcer lesion misclassified as high-risk OPMD. The large area of ulcer misclassified by the algorithms was also easily misdiagnosed by the dentists due to the similar size of the lesion. Another high-risk OPMD case [Fig. 10(c)] is wrongly identified as cancer. In fact, there is an intrinsic difficulty in distinguishing high-risk OPMD from cancer in some cases, which is why a biopsy is often needed for further confirmation. This can be considered less critical than the first example, as the misclassified lesion was either cancerous or precancerous in nature. The reticular oral lichen planus image [shown in Fig. 10(d)], which suffered with reflective light, shows similar characteristics to normal tissue, and it is underdiagnosed as normal by the deep learning algorithm. In addition, the reason for all these misclassifications could be that the training data does not include many of these kinds of images. Training with more of these challenging samples is likely to boost classification performance.

Our study does have some limitations. One major limitation is a retrospective study, with all of the inherent limitations. Another limitation is that the proposed method is validated on finite oral images. (1) The images of oral diseases were only obtained from one hospital, with the patients coming from surrounding cities. More data from additional hospitals and provinces are required to test the generalizability of our approach to other patient populations. (2) The images in the experiments were mainly collected from four different smartphone cameras. Although the results are promising, the proposed method is not fully validated on other smartphone cameras. (3) There are only five categories for evaluation in our study and there are many other oral disease types not included. However, we believe the generalization performance of the model can be guaranteed after using other kinds of oral cavity images from multiple users or phones for network training, as the appearance of disease variants can be learned by the network. In addition, further validation on other oral disease types (such as oral thrush) and on various smartphone cameras is still required to fully establish the performance characteristics of our AI diagnosis system. In summary, our algorithm may be helpful in places where the doctor has less experience or for patient self-prediagnosis, but is unlikely to become a tool for clinical specialists.

We will continue to collect more oral cavity images for future studies. We believe that more data will improve results significantly, as large datasets are key to deep learning algorithms. Meanwhile, our focus will be on how to combine the appearance characteristics of lesions and the powerful learning ability of convolutional neural networks to classify captured images more robustly, effectively, and accurately.

## 5 Conclusions

In this study, we address the challenge of automatically identifying oral disease in smartphone-based white light images and present a simple yet effective image-collection approach and resampling method that leverages the advantages of deep learning algorithms. We evaluate the performance of the recent HRNet, which is pretrained on the ImageNet, on our collected images with five categories of disease. The results demonstrate that our methods can effectively improve the prediction performance on early cancer diagnosis using smartphone photographic image.

## Code, Data, and Materials Availability

The source code is available at https://github.com/hplin6/oral_disease_diagnosis. Data and materials underlying the results in this paper are not publicly available at this time but may be obtained from the authors upon reasonable request.
